# Comparative analysis of fungal communities between herbicide-resistant and -susceptible *Alopecurus aequalis*

**DOI:** 10.3389/fcimb.2022.1094853

**Published:** 2022-12-23

**Authors:** You Zhan, Haozhe Liu, Ziheng Cao, Wen Chen, Zongfang Li, Lianyang Bai, Lang Pan

**Affiliations:** College of Plant Protection, Hunan Agricultural University, Changsha, China

**Keywords:** ALS, mesosulfuron-methyl, *Alopecurus aequalis*, non-target site resistance, glutathione S-transferase (GST), fungi

## Abstract

**Introduction:**

*Alopecurus aequalis* is a grass species invading Chinese canola and wheat fields. An *A. aequalis* KMN-R population surviving mesosulfuron-methyl treatment with recommended rates was acquired from wheatland. Here, we aimed to confirm the resistance profiles of KMN-R to acetolactate synthetase (ALS) inhibiting herbicides and explore the possible resistance mechanisms to mesosulfuron-methyl in this weed population.

**Methods:**

The dose-response tests performed in our study were used to test the toxicity of *A. aequalis* to ALS-inhibiting herbicides. Sanger sequencing was used to analyze the ALS gene of mesosulfuron-methyl -resistant and -susceptible *A. aequalis*. RNA sequencing analysis was used to find candidate genes that may confer metabolic resistance to the mesosulfuron-methyl in resistant *A. aequalis* population. Mesosulfuron-methyl -resistant and -susceptible *A. aequalis* populations fungal composition was measured via Illumina MiSeq Sequencing.

**Results:**

Dose-response results indicated that KMN-R population evolved resistance to mesosulfuron-methyl and other tested ALS-inhibiting herbicides. Known resistance-conferring Trp-574-Leu gene mutation in *A. aequalis* ALS was detected in the KMN-R population. Pretreatment with 4-chloro-7-nitrobenzoxadiazole reversed mesosulfuron-methyl resistance in KMN-R. Glutathione S-transferases (GST) gene *GSTZ2* and *GSTT3* were highly expressed in KMN-R population. In addition, we evaluated the alpha diversity in A. aequalis, centering on OTU abundance, equality, and multiplicity, and found that the fungal community composition had more unexplained variance between KMN-R and KMN-S *A. aequalis*. We also observed higher abundances of specific fungi in KMN-R *A. aequalis*.

**Discussion:**

The results proved that resistance to mesosulfuron-methyl in A. aequalis KMN-R population is probably caused by target site- and non-target site-based relating GST and provided the basis for further research between fungal interaction and herbicide resistance.

## 1 Introduction

*Alopecurus aequalis* is an annual winter grass weed widespread in the temperate zone of north earth (Hashim et al., 2012). *A. aequalis* can germinate across a broad range of environment terms, making it a serious predominant weed in several Chinese wheat and canola fields (Zhao et al., 2018a). Strong stooling of *A. aequalis* strengthens its competitive capacity with crops, reducing crop productivity (Zhao et al., 2018a). Universal methods practicable for controlling *A. aequalis* still rely significantly on herbicides, particularly acetolactate synthetase (ALS)-inhibiting herbicides. Plant ALS is a crucial enzyme for branched-chain amino acid biosynthesis (Durner et al., 1991). ALS-inhibiting herbicide has been wildly used to control weeds. Nevertheless, more and more weeds are resistant to ALS-inhibiting herbicides due to their extensive use. So far, ALS-inhibiting herbicide resistance has been reported in 166 weeds (Heap, 2022). Figuring out the resistance mechanisms is significant to develop valid treatment techniques to slow down the weeds’ developing resistance.

Herbicide resistance mechanisms can be divided into two types: target site resistance (TSR) and non-target site resistance (NTSR) (Powles and Yu, 2010). TSR can just simply be confirmed through exploring target site gene mutations or overexpression. For ALS, amino acid changes at eight ALS gene sites (Ala122, Pro197, Ala205, Asp376, Arg377, Trp574, Ser653, and Gly654) evolve ALS-inhibiting herbicide resistance in various weeds (Powles and Yu, 2010; Yu and Powles, 2014b). Different from TSR, NTSR results from mechanisms reducing herbicides to reach the target site (Délye et al., 2013). Metabolic resistance is a predominantly known NTSR so far, though it is still in the process of being studied as related to multiple gene families, such as glutathione S-transferases (GSTs), ATP-binding cassette (ABC) transporters, cytochrome P450 monooxygenases (P450s), and glucosyltransferases (GTs) (Yuan et al., 2007). Recently, metabolic resistance to ALS-inhibiting herbicides was increasingly reported in some weed species, for instance, *Echinochloa crus-galli* (Riar et al., 2013), *Amaranthus tuberculatus* (Shergill et al., 2018), *Amaranthus palmeri* (Nakka et al., 2018), *Echinochloa phyllopogon* (Iwakami et al., 2014), *Bromus rigidus* (Owen et al., 2012), *Beckmannia syzigachne* (Wang et al., 2022), and *Myosoton aquaticum* (Bai et al., 2019).

Mesosulfuron-methyl is an ALS-inhibiting herbicide commonly applied to control grassy weed. Long-standing usage of mesosulfuron-methyl makes it less effective. Lately, mesosulfuron-methyl susceptibility in *A. aequalis* also decreased (Guo et al., 2015b). As documented, known TSR mutation sites of ALS in *A. aequalis*, such as Pro-197-Arg/Ser/Tyr/Thr and Trp-574-Leu, responsible for mesosulfuron-methyl resistance, have been characterized (Guo et al., 2015a; Xia et al., 2015; Guo et al., 2018; Zhao et al., 2019b). Although metabolic resistance has been recognized in mesosulfuron-methyl-resistant *A. aequalis*, identification of resistance-related metabolic enzyme gene has proceeded slowly. Three CYP450 contigs (*CYP71A4*, *CYP94A1*, and *CYP709C56*), one GST contig (*GSTU1*), and one GT contig in *A. aequalis* were reported to be involved in mesosulfuron-methyl resistance (Zhao et al., 2019a; Zhao et al., 2019b; Zhao et al., 2022), and only the *CYP709C56* was explicitly identified to have considerable effect, endowing metabolic herbicide resistance in *A. aequalis* (Zhao et al., 2022).

Plants form mutualistic symbioses with fungi in natural systems (Rodriguez and Redman, 2008). Adaptability benefit given by fungi expressing mutualistic lifestyles contained abiotic and biotic stress tolerances, enhanced reproduction, and promoted growth (Yan et al., 2019; Ganie et al., 2022). Plants under stress conditions will employ a “cry for help” strategy, producing “chemical messengers” to activate microbial endophytes to alleviate the stress-induced damage (Liu et al., 2020a). Fungi, such as *Epichloë coenophiala* in *Lolium arundinaceum* and *Epichloë* in *Hordeum brevisubulatum*, are capable of tolerating abiotic stress (Dinkins et al., 2017; Chen et al., 2021). Currently, in weeds, only endophytes in *Polypogon fugax* enhance quizalofop-p-ethyl resistance (Liu et al., 2020b). Bioactive secondary metabolites, particularly bio-synthesizing using fungi, have been found to be promising new compounds to exploit in agriculture for use in controlling weeds (Macías-Rubalcava and Garrido-Santos, 2022).

In 2019, farmers in Anhui province discovered that the mesosulfuron-methyl-recommended rate cannot control *A. aequalis* in the wheat field. Thus, the objectives of the present study were to 1) confirm the resistance level to mesosulfuron-methyl in putative resistant *A. aequalis* populations; 2) investigate the TSR mechanism in mesosulfuron-methyl-resistant *A. aequalis* populations; 3) investigate the NTSR mechanisms in mesosulfuron-methyl-resistant *A. aequalis* populations; and 4) investigate and quantify the fungi between the mesosulfuron-methyl-resistant and -susceptible *A. aequalis* populations.

## 2 Materials and methods

### 2.1 Plant material and chemicals

The resistant *A. aequalis* population (KMN-R) was gathered in the Chinese Anhui wheat field where mesosulfuron-methyl was applied for at least 5 years. The susceptible population (KMN-S) was gathered from the vacant field where mesosulfuron-methyl was never applied. Seeds from 60 mature seedlings were gathered stochastically manually in each population. All collected seeds were dried and preserved in plastic bags until use.

Herbicides applied in the response test were shown in **Table S1**. GST inhibitor 4-chloro-7-nitrobenzoxadiazole (NBD-Cl, 97%) and cytochrome P450 inhibitor malathion (95%) were bought from Sigma.

### 2.2 Whole plant response to acetolactate synthetase-inhibiting herbicides

About 30 A*. aequalis* seeds were seeded in each pot containing nutrient soil, and the pot was moved to artificial climate chambers with a 14-h day of 20°C and 10-h night of 15°C at 75% humidity. After growing for 4 weeks, seedlings were thinned to 20 in each pot and sprayed with tested ALS-inhibiting herbicides (**Table S1**) and returned to the greenhouse. After treating for 3 weeks, aboveground seedlings were cut to determine the fresh weight.

### 2.3 Cloning and sequencing of *A. aequalis ALS* genes

Fresh leaf tissue (100 mg) in each plant was used to extract genomic DNA (gDNA) using a Tiangen Biotech Co. DNA Kit with instructions. Primers (**Table S2**) were designed and used to amplify *A. aequalis ALS* gene fragment involving all known ALS-inhibiting herbicide resistance-related mutations. Polymerase chain reaction (PCR) was conducted, and the PCR run was set as described (Wang et al., 2022). Amplified PCR products were also purified, cloned, and sequenced as described (Wang et al., 2022). Ten plants randomly selected from each population were used for sequencing. Sequences were aligned using BioEdit sequence alignment editor software and DNAMAN.

### 2.4 Effects of glutathione S-transferases and cytochrome P450 inhibition on the resistance to mesosulfuron-methyl

Seeds from *A. aequalis* populations were cultured by following the same method as above. *A. aequalis* seedlings were sprayed with mesosulfuron-methyl, NBD-Cl, NBD-Cl plus mesosulfuron-methyl, malathion, and malathion plus mesosulfuron-methyl. NBD-Cl (270 g a.i. ha^-1^) was used 48 h before mesosulfuron-methyl treatment, and malathion (1,000 g a.i. ha^-1^) at 2 h. Mesosulfuron-methyl was applied following the doses as above. Twenty-one days after mesosulfuron-methyl application, photographs were recorded for growth status and aboveground weight per pot was determined.

### 2.5 RNA sequencing data

TaKaRa Biotech RNAiso Plus was used to extract total RNA from three KMN-R and three KMN-S seedlings with instructions. NanoPhotometer^®^ spectrophotometer was used to analyze the quantity and quality of total RNA. RNA sequencing (RNA-seq) was performed using Illumina platform to generate paired-end reads. Data from KMN-R and KMN-S seedlings were merged to assemble with Trinity (Grabherr et al., 2011) for generating the reference transcriptome. The clean read was filtered from raw read for downstream analyses, and clean data were mapped to reference for obtaining each gene read count. The gene read count for each transcript was normalized to fragments per kilobase of exon per million fragments mapped (FPKM). The expression level of each gene was estimated using RNASeq by expectation maximization (RSEM) (Li and Dewey, 2011). Differential expression between KMN-R and KMN-S was compared using *t*-test (*p* < 0.05). Differentially expressed genes (DEGs) were analyzed for Gene Ontology (GO) enrichment using GOseq R packages (Young et al., 2010).

### 2.6 Gene expression analysis of NTSR contigs in *A. aequalis*

The candidate NTSR contig was chosen based on the following criteria: upregulated for >2-fold in KMN-R than KMN-S (*p* < 0.05) and related to GST, ABC transporter, and GT annotation. Samples provided for RNA-seq were used to validate GST, ABC transporter candidate, and GT contig using quantitative real-time PCR (qRT-PCR). Primers (**Table S3**) for GST, ABC transporter, and GT contig were designed using an online software (https://sg.idtdna.com/scitools/Applications/RealTimePCR/). The eukaryotic translation initiation factor 4A (*eIF4A*) gene was used as an internal control in *A. aequalis* (Zhao et al., 2018b). Only a particular PCR band was amplified in each primer, and the negative control did not amplify anything. The amplified PCR product was sequenced to confirm the anticipated gene. qRT-PCR was performed using ABI-7500 Fast Real-Time PCR System with TaKaRa SYBR^®^ Premix Ex Taq™ kits. Primer efficiencies of all studied genes were 94%–115%. qRT-PCR experiments and expression level analyses were conducted as described (Pan et al., 2019).

### 2.7 Statistical analyses

Each treatment contained three replicates, and every experiment was repeated twice. Herbicide effective rates reducing 50% plant inhibition in fresh weight (GR_50_) were calculated using four-parameter log-logistic curve for fitting in SigmaPlot software as described (Wang et al., 2022). Resistance indexes (RIs) were determined as the GR_50_ of KMN-R divided by the GR_50_ of KMN-S to indicate resistant levels for the KMN-R population.

### 2.8 DNA extractions and Illumina MiSeq sequencing preparation in *A. aequalis*

To remove surface fungi in *A. aequalis*, sterile water, 70% alcohol, and sodium hypochlorite solution (with 2.5% active Cl^-1^) were used to separate soil from seedlings, then sterile water was used to wash the seedlings five times, and sterile absorbent papers were used to remove water. Ultimately, seedlings were cut into small fragments using flame-sterilized scalpels and stored at -80°C until use. All procedures were conducted under a sterile environment. Power Soil DNA Isolation Kit was used to extract microbial DNA from 1.0 g *A. aequalis* (wet weight). Primer pairs ITS1F (5’-CTTGGTCATTTAGAGGAAGTAA-3’) and ITS2R (5’-GCTGCGTTCTTCATCGATGC-3’) were used to generate fungal internal transcribed spacer (ITS) amplicon libraries. PCR was conducted, and the PCR run was set as described (Wang et al., 2019). Amplified PCR products were purified on 2% agarose gel. Illumina MiSeq platform was used to paired-end sequence (2 × 300 bp for fungi) purified amplicons by Majorbio Bio-Pharm Technology Co. Ltd. (Shanghai, China). Data were analyzed using Majorbio I-Sanger Cloud Platform (www.i-sanger.com).

### 2.9 Bioinformatic processing in *A. aequalis*

Trimmomatic (Bolger et al., 2014) and FLASH (Magoč and Salzberg, 2011) were used to demultiplex, quality-filter, and merge raw fastq files with the following criteria: (a) Reads cut at any site can receive average quality scores <20 over 50-bp moving windows. (b) Primers used were correctly matched, permitting two nucleotide mismatches, and a read involving a blurred base was removed. (c) Sequences with an overlap of >10 bp were merged on account of the overlapped parts. Then, the remaining distinct sequences were calculated for pairwise distance and created a distance matrix. A 0.03 operational taxonomic unit (OTU) definition (97% sequence similarity cutoff level) was used to cluster OTUs using UPARSE, and OTUs contained multitude unity taxonomy. Singleton was removed from the dataset to minimize the effect of sequencing artifacts (Dickie, 2010). UCHIME (Edgar et al., 2011) was used to identify and remove chimeric sequence. The RDP Classifier algorithm against the UNITE v.7 ITS database (fungi) was used to analyze the taxonomy of ITS sequences with a confidence threshold of 70%.

## 3 Results

### 3.1 Dose–response to acetolactate synthetase-inhibiting herbicides

**Figure 1:**
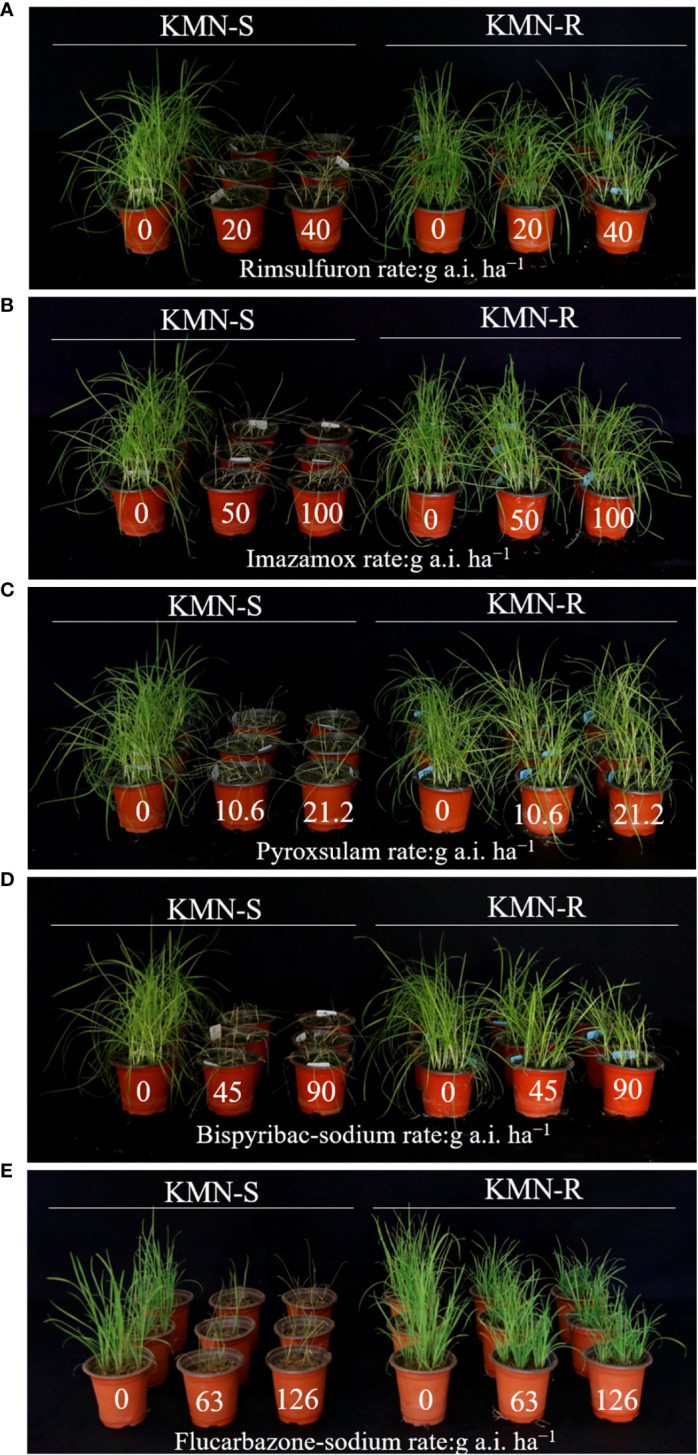
Sensitivities of the susceptible (KMN-S) and resistant (KMN-R) *Alopecurus aequalis* populations to different ALS-inhibiting herbicides. **(A)** Rimsulfuron. **(B)** Imazamox. **(C)** Pyroxsulam. **(D)** Bispyribac sodium. **(E)** Flucarbazone sodium. If seedlings grew well after herbicide application, they were regarded as resistant; if seedlings suffered severe damage or death, they were regarded as sensitive. The numbers mean herbicide application rates (g a.i. ha^−1^).

Whole plant response tests showed that the KMN-R population evolved resistance to mesosulfuron-methyl (**Table 1**). The GR_50_ value of KMN-R was 131.03 g a.i. ha^−1^, while the GR_50_ value of KMN-S was 1.14 g a.i. ha^−1^. The estimated RI for KMN-R was 114.94-fold by dividing the GR_50_ value of KMN-R to that of KMN-S, indicating high-level mesosulfuron-methyl resistance. KMN-S was susceptible to five tested ALS-inhibiting herbicides (**Figure 1**). Compared with KMN-S, the resistant KMN-R endowed resistance to the five ALS-inhibiting herbicides, including rimsulfuron, imazamox, pyroxsulam, bispyribac-sodium, and flucarbazone-sodium (**Figure 1**).

**Table 1:** Influences of GST inhibitor (NBD-Cl) and cytochrome P450 inhibitor (malathion) on *Alopecurus aequalis* growth to mesosulfuron-methyl.

Populations	Treatments	GR_50_ (g a.i.ha^-1^) (SE)^a^	RI^b^
KMN-R	NBD-Cl+ mesosulfuron-methyl	47.16 (13.77)	41.37
	Malathion+ mesosulfuron-methyl	156.44 (58.69)	137.23
	mesosulfuron-methyl	131.03 (21.06)	114.94
KMN-S	NBD-Cl+ mesosulfuron-methyl	1.10 (0.20)	0.96
	Malathion+ mesosulfuron-methyl	1.03 (0.11)	0.90
	mesosulfuron-methyl	1.14 (0.50)	1

a SE, standard error. GR_50_, herbicide effective rates reducing 50% plant inhibition in fresh weight.

b RIs, Resistance Indexes.

### 3.2 Comparison of *ALS* gene sequences

**Figure 2:**
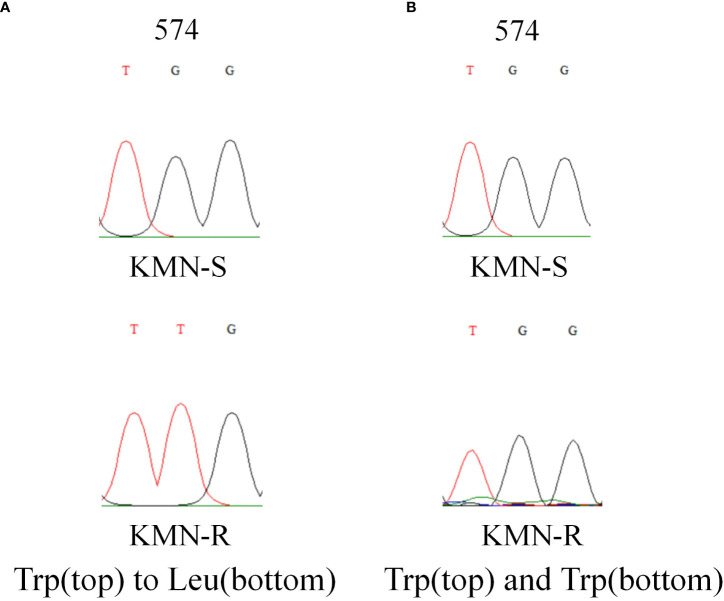
DNA sequencing revealed **(A)** Trp-574-Leu change in *ALS1* and **(B)** no change in *ALS2* in KMN-R *Alopecurus aequalis* compared with KMN-S. The red and black lines mean the signal intensities of the “T” and “G” bases in Sanger sequencing. *ALS1* and *ALS2* is the two copies of *ALS* gene.

The length of the *A. aequalis ALS* partial gene sequences was 1,917 bp. Sequence alignment between KMN-R and KMN-S samples assumed the TGG to TTG nucleotide variation, causing Trp-574-Leu mutation in KMN-R (**Figure 2**), confirming that this known resistance-conferring substitution existed in the KMN-R population.

### 3.3 Effects of glutathione S-transferases and cytochrome P450 inhibition on the resistance to mesosulfuron-methyl

Malathion at 1,000 g a.i. ha^-1^ or NBD-Cl at 270 g a.i. ha^-1^ did not influence the development of *A. aequalis* seedlings. NBD-Cl or malathion had no impact on the susceptibility of the plants in susceptible population KMN-S to mesosulfuron-methyl (**Table 1**). Treatment with malathion did not increase the toxicity of mesosulfuron-methyl to plants in resistant population KMN-R (**Table 1**). However, treatment with NBD-Cl combined with mesosulfuron-methyl increased mesosulfuron-methyl toxicity to plants in KMN-R (**Table 1**), with a markedly decreased GR_50_ value from 131.03 to 47.16 g a.i. ha^-1^.

### 3.4 Identification of differentially expressed contigs in *A. aequalis*

Owing to the absence of the *A. aequalis* genome, the reference transcriptome in *A. aequalis* assembled the sequencing data from KMN-R and KMN-S. Then, 36,996 supposed genes with contig N50 size of 1,969 bp were identified from 42,875 transcripts. Assembled sequences were annotated using SwissProt, NCBI non-redundant protein sequences (Nr), protein family (PFAM), clusters of orthologous groups of proteins (KOG), GO, and KEGG ortholog (KO) databases (**Table S4**). Based on FPKM at *t*-test (*p* < 0.05), DEGs in the KMN-R and KMN-S were assessed. A total of 1,484 differentially expressed contigs between KMN-R and KMN-S were confirmed (**Figure S1**). DEG functions were evaluated using GO enrichment analyses, and metabolic process and cellular process were significantly enriched (**Figure S2**).

Seven upregulated contigs of >2-fold (*p* < 0.05) in KMN-R than KMN-S in relation to GST, GT, and ABC transporter annotations were validated using prepared RNA-seq samples (**Table 2**). Two contigs (TRINITY_DN14492_c0_g1 with a *GSTZ2* annotation and TRINITY_DN438_c0_g1 with a *GSTT3* annotation) were markedly upregulated using qRT-PCR, fold changes of which displayed no differences with RNA-seq results (**Table 2**). These two highly expressed GST contigs appear to be involved in mesosulfuron-methyl resistance in KMN-R.

**Table 2:** Upregulated contigs annotated to metabolism identified by RNA-seq and qRT-PCR in *Alopecurus aequalis*.

Definition	Contig name	Function annotation	log_2_FC^a^	FC^b^	*t*-test
Glutathione S-transferase	TRINITY_DN14492_c0_g1	Glutathione S-transferase Z2	2.41	11.89	0.04
	TRINITY_DN438_c0_g1	Glutathione S-transferase T3	8.92	34.19	0.02
ABC transporter	TRINITY_DN3737_c0_g1	ABC transporter C family member 8	2.26	1.42	0.42
	TRINITY_DN4636_c0_g1	ABC transporter G family member 43	5.22	0.21	0.01
	TRINITY_DN5711_c0_g2	ABC transporter C family member 14	2.05	1.07	0.91
Glucosyl transferase	TRINITY_DN1491_c0_g3	Glycosyltransferase family 17	2.03	1.19	0.24
	TRINITY_DN2_c0_g3	Glycosyltransferase family 20	2.62	1.48	0.30

a Result from RNA-seq.

b Result from qRT-PCR.

FC, Fold change.

### 3.5 Richness of fungal species in *A. aequalis*

**Figure 3:**
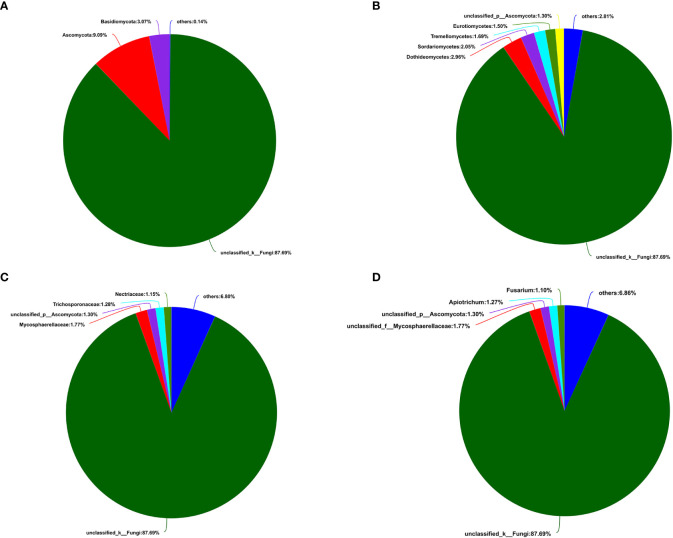
Compositions and relative plenties of fungal endophytes in *Alopecurus aequalis* on diverse taxonomic levels. **(A)** Phylum. **(B)** Class. **(C)** Family. **(D)** Genus.

A total of 405,386 fungal sequences with 290 bp average length were obtained after filtering and qualifying raw reads. A total of 155 A*. aequalis* fungal OTUs with 97% similarity were inferred in 5 phyla, 19 classes, 37 orders, 67 families, and 90 genera (**Table S5**). At the level of the phylum of *A. aequalis* fungal, the fungal community was dominated by *Ascomycota* (relative abundance 9.09%), *Basidiomycota* (relative abundance 3.07%), and *Glomeromycota* (relative abundance 0.12%) (**Figure 3A**). At the level of the class of *A. aequalis* fungal, the community was dominated by *Dothideomycetes* (relative abundance 2.96%), *Sordariomycetes* (relative abundance 2.05%), *Tremellomycetes* (relative abundance 1.69%), *Eurotiomycetes* (relative abundance 1.50%), and unclassified *Ascomycota* (relative abundance 1.30%) (**Figure 3B**). At the level of the family of *A. aequalis* fungal, the fungal community was dominated by *Mycosphaerellaceae* (relative abundance 1.77%), unclassified *Ascomycota* (relative abundance 1.30%), *Trichosporonaceae* (relative abundance 1.28%), and *Nectriaceae* (relative abundance 1.15%) (**Figure 3C**). At the level of the genus of *A. aequalis* fungal, the fungal community was dominated by unclassified *Mycosphaerellaceae* (relative abundance 1.77%), unclassified *Ascomycota* (relative abundance 1.30%), *Apiotrichum* (relative abundance 1.27%), and *Fusarium* (relative abundance 1.10%) (**Figure 3D**).

### 3.6 Diversity of fungal species between KMN-R and KMN-S *A. aequalis*

**Figure 4:**
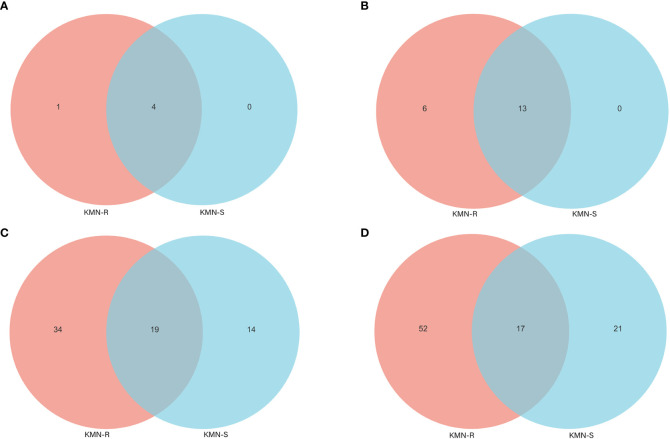
Venn diagram of fungal endophyte communities in KMN-R and KMN-S of *Alopecurus aequalis* at diverse taxonomic levels. **(A)** Phylum. **(B)** Class. **(C)** Family. **(D)** Genus.

The alpha diversity indices (Simpson and Shannon) computed from fungal OTUs of KMN-R and KMN-S *A. aequalis* populations indicated that less varied fungal OTUs were found in KMN-S than KMN-R (**Table S6**). At the phylum level of *A. aequalis* fungal, ANOVA was used to evaluate all detected phyla to test the relative abundance (%) of KMN-R and KMN-S (**Figure 4A**). Fungal community in KMN-R and KMN-S *A. aequalis* populations was dominated in four phyla, including *Ascomycota*, *Basidiomycota*, and *Glomeromycota*. It was observed that *Ascomycota* in KMN-R was significantly more abundant (*p* < 0.05) than that in KMN-S at the phylum level (**Table 3**). Particularly, *Mortierellomycota* was only found with a high relative abundance in the KMN-R *A. aequalis*.

**Table 3:** Relative plenties percentage of sequence number in common phyla between KMN-R and KMN-S of *Alopecurus aequalis*.

Phylum	R + S (%)	R (%)	S (%)
*Ascomycota*	9.10	7.93	1.17
*Basidiomycota*	3.07	1.76	1.31
*Glomeromycota*	0.12	0.10	0.02
unclassified_k:Fungi	87.71	40.20	47.51

**Figure 5:**
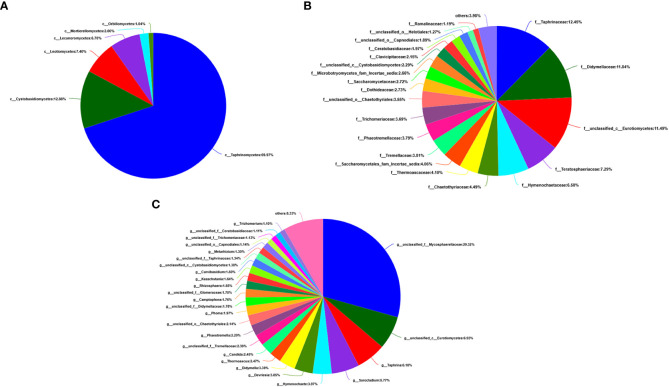
Compositions and relative plenties of unique fungal endophytes in KMN-R of *Alopecurus aequalis* on diverse taxonomic levels. **(A)** Class. **(B)** Family. **(C)** Genus.

At the level of the class of *A. aequalis* fungal, ANOVA was used to evaluate all detected classes to test the relative abundance (%) of KMN-R and KMN-S (**Figure 4B**). The fungal community in KMN-R and KMN-S *A. aequalis* populations was dominated by 13 classes. Significant enrichments (*p* < 0.05) were observed in *Dothideomycetes*, *Eurotiomycetes*, and unclassified *Ascomycota* between the KMN-R and KMN-S *A. aequalis* at the class level (**Table 4**). Specifically for *Taphrinomycetes*, *Lecanoromycetes*, *Mortierellomycetes*, *Cystobasidiomycetes*, *Leotiomycetes*, and *Orbiliomycetes*, we only observed a high relative abundance in the KMN-R *A. aequalis* (**Figure 5A**).

**Table 4:** Relative plenties percentage of sequence number in common classes between KMN-R and KMN-S of *Alopecurus aequalis*.

Class	R + S (%)	R (%)	S (%)
*Agaricomycetes*	0.37	0.32	0.05
*Dothideomycetes*	2.98	2.91	0.07
*Eurotiomycetes*	1.51	1.29	0.23
*Glomeromycetes*	0.12	0.10	0.02
*Malasseziomycetes*	0.03	0.01	0.03
*Microbotryomycetes*	0.13	0.12	0.02
*Pezizomycetes*	0.04	0.00	0.04
*Saccharomycetes*	0.70	0.42	0.28
*Sordariomycetes*	2.06	1.51	0.55
*Tremellomycetes*	1.70	1.00	0.70
unclassified_k:Fungi	88.27	40.46	47.81
unclassified_p_*_Ascomycota*	1.31	1.30	0.01
unclassified_p:*Basidiomycota*	0.78	0.25	0.53

**Figure 6:**
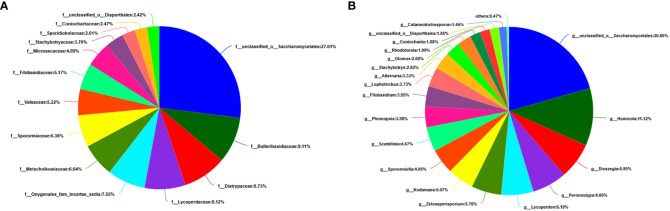
Compositions and relative plenties of unique fungal endophytes in KMN-S of *Alopecurus aequalis* on diverse taxonomic levels. **(A)** Family. **(B)** Genus.

At the level of the family of *A. aequalis* fungal, ANOVA was used to evaluate all detected families to test the relative abundance (%) of KMN-R and KMN-S (**Figure 4C**). Fungal community in KMN-R and KMN-S *A. aequalis* populations was dominated in 19 families. Significant enrichments (*p* < 0.05) were observed in *Mycosphaerellaceae* and unclassified *Ascomycota* between the KMN-R and KMN-S *A. aequalis* at the family level (**Table 5**). In addition, 34 families were only observed with a high relative abundance in the KMN-R *A. aequalis*, including *Taphrinaceae*, *Didymellaceae*, and unclassified *Eurotiomycetes* (**Figure 5B**), and 14 families were only observed with a high relative abundance in the KMN-S *A. aequalis*, including *Taphrinaceae*, *Didymellaceae*, unclassified *Saccharomycetales*, *Bulleribasidiaceae*, and *Diatrypaceae* (**Figure 6A**).

**Table 5:** Relative plenties percentage of sequence number in common families between KMN-R and KMN-S of *Alopecurus aequalis*.

Family	R + S (%)	R (%)	S (%)
*Aspergillaceae*	0.18	0.07	0.11
*Chaetomiaceae*	0.24	0.10	0.14
*Cladosporiaceae*	0.18	0.18	0.00
*Dipodascaceae*	0.26	0.18	0.09
*Glomeraceae*	0.13	0.11	0.02
*Herpotrichiellaceae*	0.09	0.05	0.05
*Hypocreales*_fam_Incertae_sedis	0.36	0.36	0.00
*Lasiosphaeriaceae*	0.02	0.01	0.01
*Malasseziaceae*	0.03	0.01	0.03
*Mycosphaerellaceae*	1.85	1.85	0.00
*Nectriaceae*	1.20	0.95	0.25
*Piskurozymaceae*	0.04	0.02	0.02
*Pleosporaceae*	0.07	0.05	0.03
*Pyronemataceae*	0.04	0.01	0.04
*Trichocomaceae*	0.21	0.18	0.03
*Trichosporonaceae*	1.34	0.72	0.62
unclassified_k:Fungi	91.58	41.97	49.60
unclassified_p:*Ascomycota*	1.36	1.35	0.01
unclassified_p:*Basidiomycota*	0.80	0.26	0.55

At the genus level of *A. aequalis* fungal, ANOVA was used to evaluate all detected genera to test the relative abundance (%) (**Figure 4D**). The fungal community in KMN-R and KMN-S *A. aequalis* populations was dominated by 17 genera. It was observed that unclassified *Ascomycota* in KMN-R was significantly more abundant (*p* < 0.05) than that in KMN-S at the genus level (**Table 6**). In addition, 52 genera were only observed with a high relative abundance in the KMN-R *A. aequalis*, including unclassified *Mycosphaerellaceae* and unclassified *Eurotiomycetes* (**Figure 5C**), and 21 genera were only observed with a high relative abundance in the KMN-S *A. aequalis*, including unclassified *Saccharomycetales*, *Humicola*, and *Dioszegia* (**Figure 6B**).

**Table 6:** Relative plenties percentage of sequence number in common genera between KMN-R and KMN-S of *Alopecurus aequalis*.

Genus	R + S (%)	R (%)	S (%)
*Apiotrichum*	1.37	0.74	0.62
*Aspergillus*	0.14	0.07	0.07
*Chaetomium*	0.08	0.07	0.00
*Cladosporium*	0.18	0.18	0.00
*Exophiala*	0.05	0.00	0.05
*Fusarium*	1.18	0.93	0.25
*Malassezia*	0.04	0.01	0.03
*Neonectria*	0.00	0.00	0.00
*Penicillium*	0.04	0.00	0.04
*Schizothecium*	0.02	0.00	0.01
*Solicoccozyma*	0.04	0.02	0.02
*Thermomyces*	0.22	0.19	0.03
*Trichocladium*	0.05	0.00	0.05
unclassified_f:*Dipodascaceae*	0.27	0.18	0.09
unclassified_k:Fungi	94.11	43.14	50.98
unclassified_p:*Ascomycota*	1.39	1.39	0.01
unclassified_p:*Basidiomycota*	0.83	0.26	0.56

## 4 Discussion

*A. aequalis* has evolved mesosulfuron-methyl resistance because of enhanced selective pressures applied to wheat fields in China. Since mesosulfuron-methyl was brought in, it is consumingly applied to control *A. aequalis* and other grassy weeds. Nevertheless, resistance to mesosulfuron-methyl happened in *A. aequalis* (Guo et al., 2015b), and it has been reported that high-level mesosulfuron-methyl resistance existed in *A. aequalis* populations (Guo et al., 2015a; Xia et al., 2015; Guo et al., 2018; Zhao et al., 2019b). In this study, the KMN-R population gathered from an Anhui wheat field has also developed mesosulfuron-methyl resistance. In the light of local farmer households, mesosulfuron-methyl has been applied in the wheat field where KMN-R gathered for >5 years. This result shows again that long-term usage of the same herbicide or herbicides with similar resistance mechanisms is the biggest risk factor to promote herbicide resistance evolutions (Norsworthy et al., 2012).

GST-mediated herbicide metabolism was confirmed in both weeds and crops (Cummins et al., 2011; Cummins et al., 2013; Evans et al., 2017; Nakka et al., 2017). NBD-Cl has been indicated to inhibit the expressed pi class GST in human and expressed phi (F) class GST in resistant *Alopecurus myosuroides* strongly (Ricci et al., 2005; Cummins et al., 2013). In this study, to investigate NTSR, NBD-Cl treatment decreased the resistance level of KMN-R to mesosulfuron-methyl to a great extent (**Table 1**). Especially, the consequent GR_50_ value of NBD-Cl plus mesosulfuron-methyl was higher than the GR_50_ value of KMN-S, showing a GST-participated enhanced metabolism and other resistance mechanisms presented in the KMN-R population and contributed to its resistance phenotype. Malathion is known to goal diverse P450 genes (Oliveira et al., 2017). In our study, pretreatment with malathion did not increase the toxicity of mesosulfuron-methyl to plants in resistant population KMN-R, indicating that there might not be any additional benefit from P450-mediated enhanced metabolism.

Even though enhanced herbicide metabolism has been found in several weeds, limited genes related to metabolic resistance are identified (Cummins et al., 2013; Iwakami et al., 2014; Iwakami et al., 2019). Here, *A. aequalis GSTZ2* and *GSTT3* genes were upregulated in the KMN-R population (**Table 2**). In weeds, *GSTF2* and *GSTF1* genes have major roles in herbicide resistance of *A. myosuroides* (Cummins et al., 2013) and waterhemp *(Amaranthus tuberculatus)* (Evans et al., 2017). In crops, tau glutathione transferases from *Citrus sinensis* (*CsGSTU*) overexpression evolved tolerance to fluorodifen and alachlor in tobacco (Lo Cicero et al., 2015; Lo Cicero et al., 2017), and *GSTBZ2* conducts the key last step in the biosynthesis of anthocyanins in corn (Marrs et al., 1995). These findings showed that *A. aequalis GSTZ2* and *GSTT3* also can have crucial roles in evolving mesosulfuron-methyl resistance. Notably, the resistant and susceptible *A. aequalis* populations in the present research were collected in the same province and with a distance of <3 km. Therefore, we ensured the conformance of the studied populations in regard to genetic background and decreased the “false-positive” alleles, irrelevant to metabolic resistance, due to this factor. Additional research is needed to evaluate the functions of the two GST genes in mesosulfuron-methyl resistance.

Environmental factors like soil quality are the major drivers of plant microbiome (Podolich et al., 2015; Whitaker et al., 2018). Lots of research indicated that temperature, pH, and soil salinity may affect microbial community structure markedly (Thiem et al., 2018). It was reported that infection with fungi *Neotyphodium* spp. enhanced herbicide tolerance in ryegrass (Vila-Aiub et al., 2003). In this study, we evaluated alpha diversity, centering on OTU diversity, evenness, and richness (**Tables 3**–**6**), and found that fungal community compositions had a more unclear differentiation between KMN-R and KMN-S *A. aequalis* populations including alpha diversity and taxon abundances because some phyla did not have a distinguishing variation related to herbicide resistance. Notably, we found a higher abundance of *Mortierellomycota* in the KMN-R *A. aequalis* (**Figure 6**). *Mortierellomycota* was found to be positively correlated with the pH, total organic matter, and cation exchange capacity in plants, which ultimately changed the accumulation of Cd (Shi et al., 2020), indicating that the abundance of *Mortierellomycota* in the KMN-R *A. aequalis* might reduce the accumulation of mesosulfuron-methyl. The strain should be isolated from KMN-R *A. aequalis* for further studies.

Exploring NTSR in *A. aequalis* is significant, as NTSR can result in unpredictable resistance patterns (Petit et al., 2010). Even though herbicide mixture and rotation are performed as manage solution for TSR, they may be unable to manage NTSR (Yu and Powles, 2014a). Luckily, the NTSR KMN-R can be inhibited with mesosulfuron-methyl plus NBD-Cl. Nevertheless, *A. aequalis* control requires more extensive solutions instead of only relying on herbicides. Finally, we identified the indicator OTUs and core fungi between GST-involved herbicide-resistant and -susceptible *A. aequalis*. This may give the basis for further research of herbicide resistance with microbial interaction.

In summary, we found that target site Trp-574-Leu mutation and GST-mediated non-target site evolved mesosulfuron-methyl resistance in an *A. aequalis* population. The KMN-R population showed broad-spectrum resistance to ALS-inhibiting herbicides of all five chemical families tested. Pretreatment with NBD-Cl reversed the resistance of KMN-R to mesosulfuron-methyl. Two GST genes (*GSTZ2* and *GSTT3*) were constitutively overexpressed in the KMN-R population. Endophytes can promote plant growth and enhance tolerance to abiotic stress. From this perspective, we compared the diversity of endophytic fungi between KMN-R and KMN-S *A. aequalis* and found that there are some special fungi in the KMN-R population when compared to the KMN-S population. These endophytic fungi may be involved in the resistance of the KMN-R population to mesosulfuron-methyl. Further studies are needed to verify the function of the two GST genes and figure out the role of endophytic fungi in the metabolic processes of herbicide resistance.

## Data availability statement

The data presented in the study are deposited in the NCBI Sequence Read Archive repository, accession number PRJNA901208 and PRJNA902298.

## Author contributions

YZ, HL, LB, and LP designed the experiments. YZ, HL, ZC, WC, and ZL performed the experiments. YZ, HL, and LP analyzed the data. YZ, LB, and LP wrote and revised the manuscript. All authors read and approved the final manuscript.
