# Supplementary material for: Comparative analysis of fungal communities between herbicide-resistant and -susceptible Alopecurus aequalis
Source: Front Cell Infect Microbiol. 2022 Dec 23;12:1094853. doi: 10.3389/fcimb.2022.1094853 (PMC9816403; doi:10.3389/fcimb.2022.1094853)
Supplement: Supplementary file 1 [file DataSheet_1.doc]

**Supplementary Material**

**Table S1**. Detailed information of the herbicide treatments applied in the dose-response experiments.

| Group^a^ | Herbicide | Formulation^b^ | Test doses (g a.i. ha^−1^) | |
| --- | --- | --- | --- | --- |
|  |  |  | KMN-R | KMN-S |
| SU | Mesosulfuron-methyl | 30g L^-1^ OD | 0，4.5，9，18，36，72，144 | 0，1.125，2.25，4.5，9，18，36 |
|  | Rimsulfuron | 25% WDG | 0，20，40 | 0，20，40 |
| IMI | Imazamox | 4% AS | 0，50，100 | 0，50，100 |
| TP | Pyroxsulam | 4% OD | 0，10.6，21.2 | 0，10.6，21.2 |
| PTB | Bispyribac-sodium | 100g L^-1^ SC | 0，45，90 | 0，45，90 |
| SCT | Flucarbazone-sodium | 70% WDG | 0，63，126 | 0，63，126 |

^a^ SU, sulfonylurea; IMI, imidazolinone; TP, triazolo-pyrimidine; PTB, pyrimidinyl-thiobenzoate; SCT, sulfonyl-aminocarbonyl-triazolinone.

^b^ OD, oil dispersion; WDG, water dispersible granule; AS, aqueous solution; SC, suspension concentrate.

**Table S2**. Primers used for the amplification of ALS genes in this study.

| Primers | Sequence (5’-3’) | Annealing temperature (°C) | Product size (bp) |
| --- | --- | --- | --- |
| ALS1-F | CAATAAAAATCTCATGCCCGT | 52.0 | 1917 |
| ALS1-R | CATGGTTCACAGTTGACCACA |
| ALS2-F | ACGCTCGCATAAAAAGCCA | 53.0 | 1917 |
| ALS2-R | GTCCTCTAGGTCGAGCTCTTGATT |
| ALS3&4-F | CACACACTCAGATAAAAAGCCA | 50.0~65.0 | 1916 |
| ALS3&4-R | AGGTCGAGCTCTTGCTGAAG |

**Table S3**. Primer sequences and amplicon characteristics of candidate genes for quantitative real-time polymerase chain reaction (qRT-PCR).

| Contig name | Primer sequences (5′→3′) | Amplicon length (bp) | Amplification efficiency (%) | Correlation coefficient |
| --- | --- | --- | --- | --- |
| TRINITY_DN14492_c0_g1 | F: TAACAGGTTCTCCCCTGTGG | 222 | 99 | 0.98 |
|  | R: ACGATTCCATGACGCATACA |  |  |  |
| TRINITY_DN438_c0_g1 | F: TACACGTTTGGATGCGGTTA | 154 | 94 | 0.98 |
|  | R: TGTCTCTCCCGAAATCAAGG |  |  |  |
| TRINITY_DN3737_c0_g1 | F: ATCAACGAGACGGAACAGTG | 104 | 115 | 0.99 |
|  | R: AGGGAATCAGTTGCACCTTC |  |  |  |
| TRINITY_DN4636_c0_g1 | F: AAACAAGAGAACTGGGACTGG | 196 | - | - |
|  | R: TCCACTGACACCAACTAATGC |  |  |  |
| TRINITY_DN5711_c0_g2 | F: GGAAGCAGGCTTAGGCTTTT | 162 | - | - |
|  | R: ATCTTCTCGCCCTTTCCAAT |  |  |  |
| TRINITY_DN1491_c0_g3 | F: GCAGTTTTTGCTTCCGCTAC | 250 | 106 | 0.99 |
|  | R: CGGTCGAAGTTCTCAAGGAG |  |  |  |
| TRINITY_DN2_c0_g3 | F: TCGTGAAGTAACCATGCTCTG | 219 | 113 | 0.99 |
|  | R: ACAATCGCCTAATCCTCTTGG |  |  |  |

**Table S4**. Sequence annotation of the *Alopecurus aequalis* transcriptome.

| Public database | Number of unigenes | Percentage (%) |
| --- | --- | --- |
| Annotated in COG | 7237 | 19.56 |
| Annotated in GO | 13760 | 37.19 |
| Annotated in KEGG | 12773 | 34.53 |
| Annotated in KOG | 10207 | 27.59 |
| Annotated in Pfam | 13597 | 36.75 |
| Annotated in Swiss-Prot | 14661 | 39.63 |
| Annotated in NR | 19889 | 53.76 |
| Annotated in at least one database | 20914 | 56.53 |

**Table S5**. The number changes of fungal endophytes in *Alopecurus aequalis* at different taxonomic levels.

| Sample | Phylum | Class | Order | Family | Genus | Species | OTU |
| --- | --- | --- | --- | --- | --- | --- | --- |
| R | 5 | 19 | 31 | 53 | 69 | 77 | 118 |
| S | 4 | 13 | 23 | 33 | 38 | 41 | 57 |
| R+S | 5 | 19 | 37 | 67 | 90 | 102 | 155 |

**Table S6**. The Alpha diversity index of the resistant and susceptible sample of *Alopecurus aequalis.*

| Sample | sobs | shannon | simpson | ace | chao | coverage |
| --- | --- | --- | --- | --- | --- | --- |
| R | 32 | 0.989275 | 0.685353 | 33.65577 | 33.26667 | 0.999944 |
| S | 17 | 0.313187 | 0.895907 | 18.15548 | 17.1 | 0.999972 |


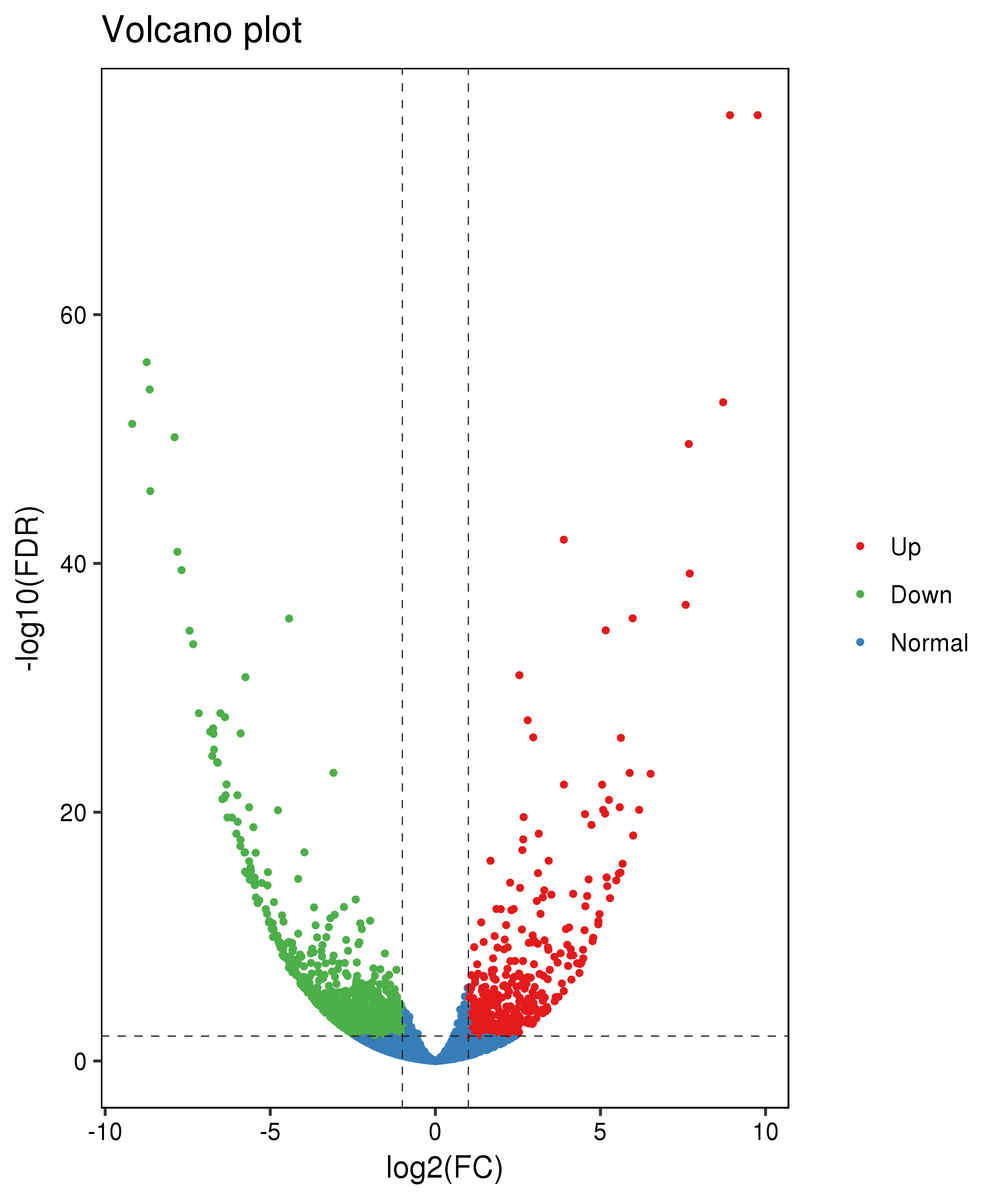


**Figure S1**. The volcano plot of differentially expressed genes (DEGs) between the susceptible (KMN-S) and resistant (KMN-R) *Alopecurus aequalis* populations. Red dots represent upregulated genes, green dots show downregulated genes, and blue dots indicate genes with no significant differences.


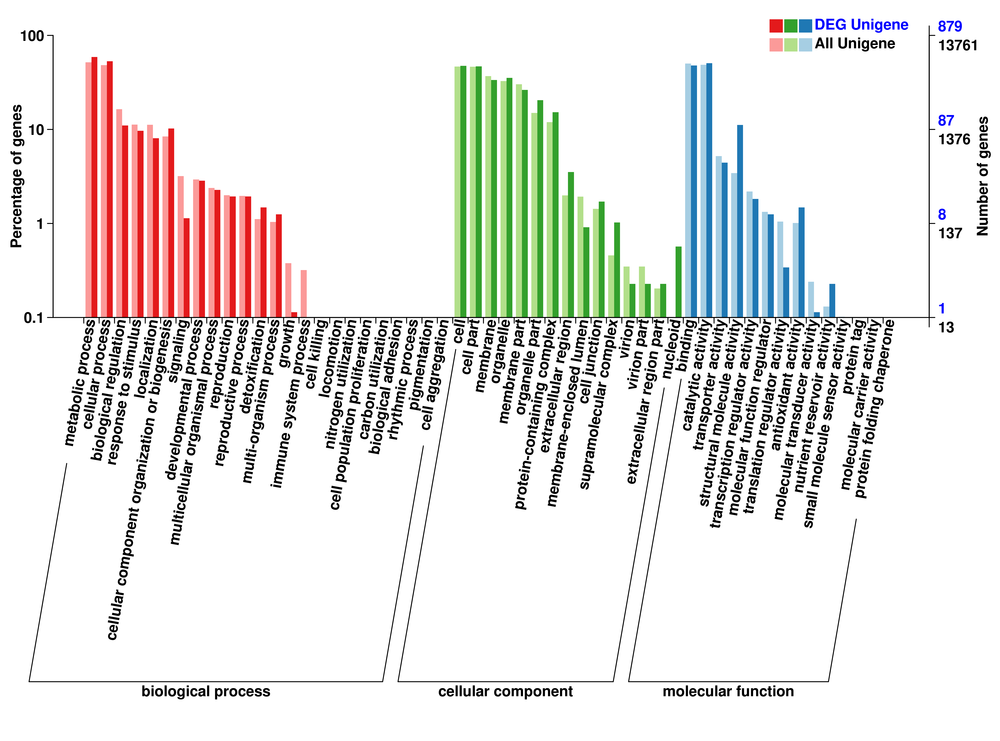


**Figure S2**. GO function classification of the all and DEGs unigene in *Alopecurus aequalis*. The unigenes were allocated to three categories: biological process, cellular component, and molecular function.
